# The association between exposure to volatile organic compounds and serum lipids in the US adult population

**DOI:** 10.1186/s12944-023-01895-z

**Published:** 2023-08-11

**Authors:** Wen-Yu Chen, Yan-Peng Fu, Hui Tu, Wen Zhong, Liang Zhou

**Affiliations:** 1 Cardiovascular Medicine Department, Nanchang University Second Affiliated Hospital, Nanchang, Jiangxi 330006 China; 2 Second Clinical Medical College of Nanchang University, Nanchang, Jiangxi 330006 China; 3 Department of Otorhinolaryngology Head and Neck Surgery, Second Affiliated Hospital of Nanchang University, No.1 Minde Road, Nanchang, China; 4 Nusring Department, Nanchang University Second Affiliated Hospital, Nanchang, Jiangxi 330006 China

**Keywords:** Volatile Organic Compounds, Lipids, NHANES, Bayesian kernel machine regression

## Abstract

**Background and Aim:**

Epidemiological evidence on the relationship between exposure to volatile organic compounds (VOCs), both single and mixed, and serum lipid levels is limited, and their relationship remains unclear. Our study aimed to investigate the associations of exposure to VOCs with serum lipid levels in the US adult population.

**Methods and results:**

The study examined the association of 16 VOC levels (2-methylhippuric acid, 3- and 4-methylhippuric acid, N-acetyl-S-(2-carbamoylethyl)-L-cysteine, N-acetyl-S-(N-methylcarbamoyl)-L-cysteine, 2-aminothiazoline-4-carboxylic acid, N-acetyl-S-(benzyl)-L-cysteine, N-acetyl-S-(n-propyl)-L-cysteine, N-acetyl-S-(2-carboxyethyl)-L-cysteine, N-acetyl-S-(2-cyanoethyl)-L-cysteine, N-acetyl-S-(3,4-dihydroxybutyl)-L-cysteine, N-acetyl-S-(2-hydroxypropyl)-L-cysteine. N-Acetyl-S-(3-hydroxypropyl)-L-cysteine, mandelic acid, N-acetyl-S-(4-hydroxy-2-butenyl)-L-cysteine, phenylglyoxylic acid and N-acetyl-S-(3-hydroxypropyl-1-methyl)-L-cysteine) with total cholesterol (TC), triglyceride (TG), low-density lipoprotein cholesterol (LDL) and high-density lipoprotein cholesterol (HDL) using data from the National Health and Nutrition Examination Survey (NHANES) between 2011 and 2015, and a total of 1410 adults were enrolled. The association was evaluated by Bayesian kernel machine regression (BKMR), multiple linear regression and weighted quantile sum (WQS) regression. In BKMR analysis, exposure to VOCs is positively correlated with levels of TC, TG, and LDL-C. However, statistical significance was observed only for the impact on TG. Our linear regression analysis and WQS regression generally support the BKMR results. Several VOCs were positively associated with serum lipid profiles (e.g., the ln-transformed level of mandelic acid (MA) displayed an increase in estimated changes of 7.01 (95% CIs: 2.78, 11.24) mg/dL for TC level), even after the effective number of tests for multiple testing (*P* < 0.05).

**Conclusions:**

Exposure to VOCs was associated with serum lipids, and more studies are needed to confirm these findings.

**Supplementary Information:**

The online version contains supplementary material available at 10.1186/s12944-023-01895-z.

## Introduction

Lipid metabolism disorder is a public health problem and an important risk factor for coronary heart disease (CHD), atherosclerosis, stroke, obesity and type 2 diabetes. The World Health Organization (WHO) reported that 2.6 million deaths were caused by elevated cholesterol [1]. The prevalence of dyslipidemia remains high and is still increasing [2]. The prevalence of dyslipidemia has reached 40.8% [3] in Chinese people and 53% in US people [4].

With the development of industrialization, an increasing amount of evidence indicates that environmental factors can affect blood lipid levels [5–7]. Animal experiments have demonstrated that air pollution can alter metabolism, oxidative stress and hormone disruption, thus leading to dyslipidemia [8]. Nguyen et al. found through the Korean National Environmental Health Survey that various pollutants, including volatile organic compounds, heavy metals, and phthalates, are associated with blood lipid levels in humans [9]. Volatile organic compounds (VOCs) are a common type of air pollutant from a variety of sources, including cigarette smoke, paint, industrial production and automobile exhaust [10]. Previous studies on VOCs have often been linked to the risk of developing several chronic diseases, such as asthma [11], lung function impairment [12] and cardiovascular disease [13], due to the mutagenic, neurotoxic, genotoxic, and carcinogenic potentials of these compounds [14–16]. Research is limited regarding the association between VOCs and serum lipid levels. Wiglusz et al. found that white male Wistar rats were exposed to VOCs for 3 months and that triglycerides decreased [17]. Liang et al. reported that exposure to acrylamide in adolescent women can increase total cholesterol levels [18]. One population-based study showed that 1,3-butadiene urinary metabolite levels were positively correlated with triglycerides and low-density lipoprotein cholesterol [19]. The results of studies are controversial, and the analyses are mainly based on single VOC models. Humans in the real world are exposed to multiple VOCs that may interact with each other.

Therefore, we hypothesized that higher concentrations of single or mixed VOCs could affect lipid profiles and it is necessary to investigate the association of single and multiple VOC exposures with selected serum lipid profiles, including total cholesterol (TC), high-density lipoprotein cholesterol (HDL), low-density lipoprotein cholesterol (LDL), and triglyceride (TG), based on the population from the Health and Nutrition Examination Survey (NHANES).

## Materials and methods

### Study participants

We used publicly accessible data from NHANES, which is a stratified, multistage probability sample representing the civilian noninstitutionalized population of the United States. The NHANES 2011–2015 survey cycle includes complete data on urinary VOC metabolites. Considering that VOC metabolites can be detected in the urine for a longer period of time than in the blood, those participants with data on urinary VOC metabolites, serum lipid profiles (TC, LDL, HDL, and TG), and confounding factors were considered eligible for inclusion in the study. Then, we obtained the necessary datasets from the NHANES website. Finally, we included a total of 1410 adults in the NHANES from 2011 to 2016.

### Serum lipid measurements

Serum lipid samples collected by CDC laboratories included TC, LDL, HDL, and TG. CDC laboratories store and process these serum samples according to a standard protocol before they are shipped to the University of Minnesota, Minneapolis, MN, for analysis. TC, LDL, HDL, and TG were measured using an enzymatic method on Roche modular P and Roche Cobas 6000 chemistry analyzers (USA 46250-0446). Serum levels of TC, LDL, HDL, and TG were analyzed using colorimetric methods. The wavelength used for TC detection was 505/694 nm. HDL-C was measured by the formation of a blue‒violet precipitate through the action of cholesterol oxidase with hydrogen peroxide, and the detection wavelength was 600 nm/700 nm. TG levels were determined by measuring the glycerol product obtained through hydrolysis by lipoprotein lipase, with a detection wavelength of 505/700 nm. LDL-C was calculated using the Friedewald equation. HDL and TG were transformed with the natural logarithm (ln) because of their right skewed distribution in this study. For detailed methods, please refer to the Laboratory Method Files on the NHANES website (https://www.cdc.gov/nchs/nhanes/index.htm).

### Urinary VOC metabolites

Ultra-performance liquid chromatography coupled with electrospray tandem mass spectrometry (UPLC-ESI/MSMS) [20] was used to quantify urine VOC metabolites (Acquity UPLC® HSS T3 Part no. 186,003,540, 1.8 μm × 2.1 mm × 150 mm, Waters Inc). The detection rates of all 16 VOC urinary metabolites in the study population were over 75%. The parent compound, aberration, detection rate and concentration range of all 16 VOC urinary metabolites are presented in Table A.1. Measurements below the limit of detection (LOD) are counted as LODs divided by the square root of 2. An enzymatic technique was used to determine urinary creatinine levels by using a Roche Cobas 600 Analyzer (University of Minnesota). Concentrations of the above 16 VOC metabolites were normalized to creatinine concentrations. The range of urinary creatinine is 4 to 481 mg/dL.

### Covariates

Based on previous studies, covariates may influence the association between VOC concentration and serum lipid levels [17–19]. Continuous variables, including age, serum cotinine, energy intake and physical activity, were treated as continuous variables. Energy intake was calculated based on self-reports of the type and amount of food consumed in the past 24 h in the NHANES dietary review. Physical activity was defined as the total minutes of weekly recreational activity and calculated by the Global Physical Activity Questionnaire (GPAQ): the number of minutes of moderate-intensity recreational activities on a typical day multiplied by the number of days of moderate-intensity recreational activities on a typical week [21]. Drinking was divided into “yes” or “no” based on the question “Have you had at least 12 drinks of any type of alcoholic beverage in any one year?” Categorical variables included sex (men, women), marital status (married or with partners, widowed or divorced, and unmarried), education level (less than high school, high school graduate/GED or equivalent, some college or associate degree, or college graduate or above), family poverty-income ratio (PIR) (low, PIR ≤ 1; middle, 1<PIR ≤ 3; high, PIR > 3), race (Mexican American, other Hispanic, non-Hispanic white, non-Hispanic black, and others), and drinking (yes, no). These variables may simultaneously influence both blood lipid levels and urinary VOC levels.

### Statistical analyses

Descriptive statistics for participant characteristics were calculated by using the NHANES-specific sample VOC weights (divided by 3), which were used to account for the complex survey design. Given the right-skewed distributions of all VOC metabolite levels, they were transformed by natural logarithm to achieve data normality. Pairwise correlations of all VOC metabolites were assessed with Spearman correlation coefficients.

Given the potential synergistic and antagonistic effects between VOCs, our study used the Bayesian kernel machine regression (BKMR) method to analyze the joint effect of multiple VOC exposures on serum lipid profiles. BKMR is also a flexible statistical algorithm suitable for analyzing the associations between a single VOC exposure and each serum lipid parameter [22]. The formula for the BKMR model is as follows:

$${Y}_{i}=h\left({\mathbf{z}}_{i}\right)+{\mathbf{x}}_{i}^{\text{T}}\varvec{\upbeta }+{?}_{i}$$

where Y_i_ represents the i-th outcome variable, Zi represents the vector of mixed exposure pollutants, X_i_^T^ represents the vector of covariates, β is the vector of regression coefficients corresponding to the covariates, and${?}_{i}$ represents the residual term.

VOC levels were converted into z scores in BKMR analysis. BKMR utilizes Markov Chain Monte Carlo (MCMC) sampling for inference. In this study, all BKMR analyses were conducted with 50,000 MCMC iterations. The BKMR algorithm design does not incorporate weighting parameters; hence, we did not consider sample weights. The contribution of each metabolite to the mixture effect is represented by the posterior probability values (PIP values) in the BKMR model. The PIP values range from 0 (least important) to 1 (most important). The BKMR model was also utilized to examine the potential interactions among VOCs by predicting serum lipid profiles while keeping all other VOC exposures at the median, 10th percentile, or 90th percentile. All BKMR models were adjusted for age, sex, race, marital status, education level, BMI, PIR, drinking, smoking, creatinine, physical activity, and energy intake. Univariate exposure-response curves for each VOC metabolite concentration and serum lipid profiles are presented with other VOC metabolite concentrations fixed at median levels. The expected change in TC-, LDL-, HDL-, and TG-associated simultaneous changes in all VOC exposures was calculated from their lowest quartile concentrations to estimate the cumulative effect of VOC exposure. Subgroup analysis was performed to further analyze, stratified by smoking status (smokers, serum cotinine > 10 ng/ml; nonsmokers, serum cotinine ≤ 10 ng/mL) [23], and explore heterogeneities in the effect of VOCs on TC, LDL, HDL, and TG.

The secondary analysis was performed using weighted multiple linear regression and weighted quantile sum (WQS) regression. All weighted multiple linear regression models were adjusted for age, sex, race, marital status, education level, BMI, PIR, drinking, smoking, creatinine, physical activity, and energy intake. Then, considering the moderate to strong correlation between VOCs, we adjusted P values by the effective number of tests (Meff) extracted by the formula below for multiple testing, where ${\lambda }_{i}$represents the eigenvalues of the similarity matrix. The adjusted *P* values were 0.005 in this study [24].

$$\left\{\begin{array}{c}{M}_{eff}=\sum _{i=1}^{M}f\left(\left|{\lambda }_{i}\right|\right)\\ f\left(x\right)=I\left(x\ge 1)\right.+\left(\text{x}-?x?\right), x\ge 0\end{array}\right.$$

The WQS regression [25] methodology is employed to evaluate the impact of chemical mixtures and ascertain the influential components within the mixture. The data were divided into a training dataset and a testing dataset in a 3:7 ratio, and 10,000 bootstrapped samples were generated in WQS. The training dataset was used to estimate the weights of the variables, while the testing dataset was employed to assess the significance of the mixture.

The survey-weighted linear regression analyses, BKMR and WQS regression were implemented in R software via the R packages “survey”, “bkmr” and “gWQS”, respectively. Statistical significance was defined as a two-tailed *P* < 0.05.

## Results

### Population characteristics

A survey-weighted description of the characteristics of the study population is shown in Table A2. A total of 1410 participants, who were mainly nonsmokers (75.3%) and nondrinkers (80.7%), had a mean age of 48.0 (17.09) years, including 726 men (51.8%). Their mean levels of TC, TG, HDL, and LDL were 193.08 mg/dL, 115.52 mg/dL, 54.94 mg/dL, and 115.04 mg/dL, respectively. The detection rates of the majority of VOCs are above 90% (Table A1). Pearson’s correlation coefficients were used to show the correlation between any two of the 16 ln-transformed VOC metabolites (Fig. A.1). The Pearson correlation coefficients ranged from − 0.01 to 0.81, and the heatmap showed a strong correlation between 3MHA + 4MHA and 2MHA (R = 0.81); the other correlations were relatively weak.

### Bayesian kernel machine regression models

**Fig. 1:**
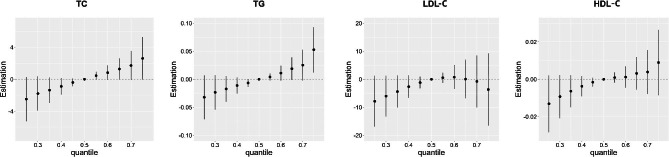
The joint effects of the VOC mixture on the serum lipid profiles estimated by Bayesian kernel machine regression (BKMR).

**Fig. 2:**
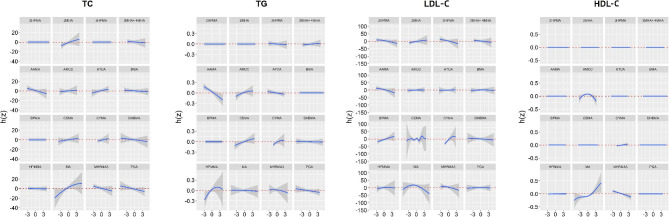
Univariate exposure–response functions and 95% credible intervals (shaded areas) for each VOC with the other VOCs holding at the median

In the mixture models, almost all serum lipids increased as the VOC mixture exposures increased (Fig. 1). For the associations of single VOCs with TC, TG, LDL, and HDL. Both 2MHA and MA were slightly positively associated with TC, and both CYMA and HPMMA were positively associated with TG. while holding the other VOC mixture components at their median values (Fig. 2). Almost all VOCs were not associated with HDL, and the trend of HDL levels showed an approximately horizontal straight line. PIP values in different models are displayed in Table A3 and AMCC had the highest PIP value for HDL-C. In addition, we did not observe significant interactions among VOCs (Figure A3-A6).

**Fig. 3:**
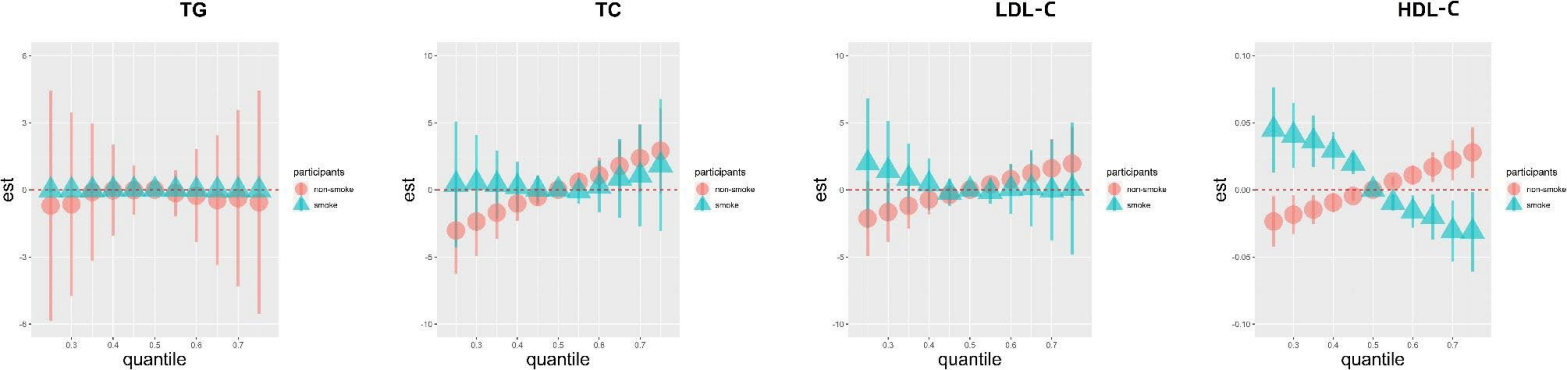
The joint effects of the VOC mixture on the serum lipid profiles estimated by Bayesian kernel machine regression (BKMR) in smokers and nonsmokers

For further investigation, stratified analysis by smoking status was performed. HDL had an upward trend for nonsmokers and the opposite trend was observed for smokers. It does appear as although there are some differences for nonsmokers and smokers in TC and LDL, although not as clear as HDL, while the trend is clearly flat for both smokers and nonsmokers in TG (Fig. 3 and Fig A.2).

### Secondary analysis

Multiple linear regression was performed as a secondary analysis to compare with our primary analysis and quantify the correlation. The specified results are summarized in Table 1. Some incongruity was found between the linear regression models and BKMR models in the covariate-adjusted model; 2MHA was positively associated with LDL, while AAMC and DHBMA were positively associated with HDL, and only MHBMA3 was negatively associated with HDL. However, the above associations were not significant after adjusting for multiple tests. In addition, the increase in TC level was associated with ATCA and MA, and MA remained significant after adjusting for multiple tests (*P* < 0.05). The metabolite MA displayed an increase in estimated changes of 7.01 mg/dL (95% CIs: 2.78, 11.24) for TC level. The increase in TG levels was associated with higher multiple VOCs, among which CEMA, CYMA, 3HPMA and HPMMA were significant after correction by multiple tests (*P* < 0.05). Each 1-unit increase in the levels of the metabolites CEMA, CYMA, 3HPMA and HPMMA corresponded to a 9.64 (95% CIs: 4.57, 14.7), 4.82 (95% CIs: 2.18, 7.46), 8.3 (95% CIs: 2.98, 13.63), and 10.78 (95% CIs: 4.54, 17.01) mg/dL increase in TG.

**Table 1:** Linear association between single urinary VOC metabolites and blood lipid parameters

VOCs	TC		TG		HDL		LDL	
	β (95% CI)	*P*	β (95% CI)	*P*	β (95% CI)	*P*	β (95% CI)	*P*
2MHA	2.15 (0, 4.29)	0.06	1.69 (-1.93, 5.3)	0.37	-0.35 (-1.24, 0.54)	0.45	**2.17 (0.47, 3.86)**	**0.02**
3MHA + 4MHA	1.57 (-0.74, 3.87)	0.19	2.77 (-0.86, 6.4)	0.15	-0.43 (-1.3, 0.44)	0.34	1.46 (-0.38, 3.29)	0.13
AAMA	-0.63 (-4.46, 3.2)	0.75	-0.09 (-6.82, 6.64)	0.98	**1.28 (0.21, 2.36)**	**0.03**	-1.87 (-5.33, 1.58)	0.3
AMCC	3.12 (-0.15, 6.4)	0.07	**7.58 (0.43, 14.73)**	**0.05**	-0.89 (-2.31, 0.54)	0.23	2.51 (-0.57, 5.59)	0.12
ATCA	**3.16 (0.11, 6.21)**	**0.05**	-1.05 (-6.27, 4.17)	0.7	0.65 (-0.47, 1.77)	0.27	2.72 (-0.03, 5.47)	0.06
BMA	0.74 (-2.55, 4.02)	0.66	**4.81 (0.24, 9.38)**	**0.05**	1.05 (-0.05, 2.16)	0.07	-1.28 (-3.99, 1.43)	0.36
BPMA	1.16 (-1.21, 3.53)	0.34	-1.44 (-4.43, 1.54)	0.35	-0.27 (-0.88, 0.34)	0.4	1.72 (-0.31, 3.76)	0.11
CEMA	2.77 (-1.04, 6.58)	0.17	**9.64 (4.57, 14.7)**	< 0.01*	-1.19 (-2.42, 0.03)	0.07	2.05 (-1.56, 5.67)	0.27
CYMA	1.41 (-0.26, 3.08)	0.11	**4.82 (2.18, 7.46)**	< 0.01*	-0.39 (-0.99, 0.2)	0.21	0.85 (-0.71, 2.4)	0.3
DHBMA	0.11 (-5.87, 6.1)	0.97	4.83 (-5.97, 15.64)	0.39	**2.59 (0.19, 4.99)**	**0.04**	-3.44 (-9.11, 2.23)	0.24
2HPMA	-0.23 (-3.47, 3.01)	0.89	-1.7 (-6.62, 3.22)	0.5	-0.1 (-1.03, 0.83)	0.83	0.2 (-2.44, 2.84)	0.88
3HPMA	2.14 (-1.93, 6.22)	0.31	**8.3 (2.98, 13.63)**	< 0.01*	-0.46 (-1.59, 0.67)	0.43	0.97 (-2.78, 4.72)	0.62
MA	**7.01 (2.78, 11.24)**	< 0.01*	8.81 (0.5, 17.13)	0.05	2.04 (0.16, 3.92)	0.04	3.22 (-0.8, 7.24)	0.13
MHBMA3	-0.12 (-3.08, 2.84)	0.94	**6.71 (1.25, 12.16)**	**0.02**	**-1.18 (-2.27, -0.09)**	**0.04**	-0.27 (-2.78, 2.24)	0.83
PGA	2.28 (-2.7, 7.26)	0.38	6.58 (-1.05, 14.22)	0.1	0.26 (-1.26, 1.78)	0.74	0.71 (-4.28, 5.71)	0.78
HPMMA	0.69 (-2.64, 4.03)	0.69	**10.78 (4.54, 17.01)**	< 0.01*	-0.57 (-2, 0.85)	0.44	-0.87 (-3.77, 2.02)	0.56

^*^, adjusted *P* < 0.05: the effective number of tests (Meff)

**Table 2:** The combined impact of VOC mixtures on blood lipid levels.

	β	*P*
TC	1.046	**0.000**
TG	1.12	0.06
LDL-C	-0.35	0.08
HDL-C	1.033	0.52

**Fig. 4:**
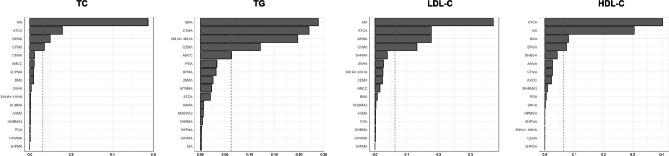
The WQS regression model estimated positive weights for each blood VOC associated with serum lipid profiles

WQS regression was used to evaluate the combined impact of VOC mixtures on blood lipid levels. We found a significant positive correlation between exposure to 16 VOCs and TR levels (β = 1.046, *P* = 0.000, Table 2). Although there was a positive correlation between VOC mixtures and levels of TC (β = 1.12, *P* = 0.06, Table 2), it did not reach statistical significance, consistent with the results of BKMR. The WQS regression weights of VOCs for serum lipid profiles are displayed in Fig. 4. For LDL-C and TC, MA had the highest weights, while BMA and ATCA were assigned the greatest weights for TR and HDL-C, respectively.

## Discussion

There have been few population studies on the association between VOCs and serum lipids, and most studies have analyzed a single VOC [18, 19, 26]. One study reported the combined effect of several VOCs in the occupational environment on serum lipids among nail technicians or petrochemical industry employees, which has limited generalizability to the general population [27, 28]. Considering the presence of multiple VOCs in the real environment, as well as the limitations of univariate analysis, our study utilized BKMR to explore the association of VOC mixture exposure with TC, TG, LDL and HDL in the general population. Specifically, with increasing levels of exposure to multiple VOCs, there was an increase in concentrations of TC, TG, and HDL, while LDL concentrations initially increased and then decreased, although only the association between VOCs and TG showed statistical significance. In the linear regression model, we also observed a positive correlation between multiple VOCs and TG levels. Similarly, in the WQS regression analysis, the VOC mixture showed a significant positive association only with TG levels. These findings suggest that TG levels are more susceptible to the effects of VOC exposure.

Interestingly, although this study identified associations between VOC exposure and serum lipids, particularly TG, through various statistical methods, the results from different statistical analyses were not entirely consistent. The PIP values in the BKMR model did not effectively identify the individual chemical substances that contributed the most to the outcomes, and the WQS model also showed discrepancies with the results from the linear regression model. These statistical methods all have their inherent limitations [29, 30], and the findings of this study need further validation in future research.

As a prevalent air pollutant, VOCs have been reported to be associated with the occurrence of diabetes [31], obesity [32], reduced lung function [33], and autoimmune diseases such as arthritis [34]. The specific mechanisms by which VOCs influence serum lipid profiles are currently not well understood [7]. This may be attributed to the decline in liver function, as the liver plays a crucial role in lipid metabolism. Consequently, hepatic cell damage can lead to various functional impairments, such as impaired LDL secretion, compromised β-oxidation, and alterations in fatty acid synthesis pathways [35]. VOCs are present in various organic solvents, and previous studies have reported their potential hepatotoxicity [36]. However, the exact mechanisms through which exposure to VOCs results in liver dysfunction remain unclear. Recent research has indicated that the major pathogenic mechanisms of VOC-induced liver dysfunction include inflammation, impaired cytochrome P450 function, mitochondrial dysfunction, and oxidative stress [37]. Previous toxicological studies on VOCs have also indicated that the mechanisms underlying VOC-induced disruption of lipid metabolism may involve inflammation, mitochondrial dysfunction, and oxidative stress [27, 38, 39]. Moreover, VOCs are a large category of pollutants, each of which has its own unique toxicity and combined toxicity and may affect serum lipid profiles through different mechanisms.

Serum lipid profiles had a certain correlation with metabolites of acrolein (CEMA and 3HPMA), crotonaldehyde (HPMMA), acrylamide (AAMA), acrylonitrile (CYMA) and styrene (MA). Feng et al.’s study showed that reducing acrolein exposure can increase high-density lipoprotein cholesterol using NHANES [26], which is consistent with our study. Two acrolein metabolites were strongly associated with TR, and compared with acrolein metabolites (3HPMA and CEMA), they seemed to have a stronger correlation with TG levels. Acrolein, as a common environmental pollutant in the US, has become a hot spot in chronic disease research. Some experimental studies [40, 41] in mice have reported that acrolein can induce lipoprotein modification and might cause dyslipidemia. Our study is the first to report the association of HPMMA (crotonaldehyde metabolite) and serum lipids. Crotonaldehyde, as a recognized harmful air pollutant, can induce oxidative stress [42] and inflammation [43] and has been confirmed to increase the risk of CVD [44], which may be related to lipid metabolism disorder. The association of acrylamide metabolite (AAMA) with TG and TC was negative and inconsistent with previous studies [18]. Zebrafish studies have also suggested that acrylamide can cause elevated TG and TC levels by modifying the function and structure of lipoproteins or enhancing the oxidation and degradation of LDL and the uptake of LDL by macrophages [45]. However, our results consider the potential synergistic and antagonistic effects between VOCs, which were not considered in previous studies. Acrylonitrile and styrene are widely used industrial organic substances, and they are similar to the other VOCs in that they produce oxidative stress and are harmful to human health [46, 47]. However, no study on the two VOCs and lipid profiles was found, and we need to conduct more research to explore these associations in the future.

The environmental sources of most VOCs are mainly produced from cigarette smoke or other industrial waste gas [48]. Interestingly, in our study, in smokers, combined VOC exposure was inversely associated with HDL. The results were reversed for nonsmokers and smokers. In addition, only nonsmokers were observed to have increased levels of the acrolein metabolites CEMA (acrolein metabolite) and HPMMA (crotonaldehyde metabolite) with increasing TG levels, which might indicate that cigarette smoke could interfere with the effect. There were only 352 smokers in our study, and the small sample size may affect the reliability of this result. A larger sample size is needed to further verify these results.

### Study strengths and limitations

There are several strengths in this study. This is the first study to investigate the associations between the combined effects of VOC exposure and serum lipid profiles in the general population from the NHANES, and the sample size was large and representative. However, several limitations should also be noted in this study. First, this study was cross-sectional and could not elucidate a causal relationship between VOC and serum lipid profiles. Second, VOC exposure data are derived from single-point urinary VOC metabolites, which might ignore the variability over time. Finally, although several potential confounders were adjusted in the analyses, the possibility of residual confounding could not be eliminated, and some confounders had not yet been collected in NHANES. Last, we did not investigate the role of VOCs in individuals with a family history of dyslipidemia and in special populations who are on long-term medication affecting lipid concentrations.

## Conclusions

This is the first study to examine the association between exposure to VOC mixtures and serum lipid profiles among the US general population. The study showed that exposure to VOCs was associated with altered lipid profiles, and the association may also be affected by smoking. In conclusion, the underlying mechanism is not yet clear, and more high-quality studies with large samples are needed.

### Electronic supplementary material

Below is the link to the electronic supplementary material.

**Additional File 1**: Table A.1 VOC metabolites targeted in this study. Table A.2 Characteristics of the study population. Fig. A.1 Pearson correlation coefficient matrix for the VOCs visualized as a heatmap. (Red shows a positive correlation, and the intensity of the shaded squares reflects the magnitude of the correlation coefficient.) Fig. A.2 Univariate exposure–response functions and 95% credible intervals (shaded areas) for each VOC in smokers and nonsmokers with the other VOCs holding at the median. Table A.3 PIP values in BKMR. 
