# Supplementary material for: The association between exposure to volatile organic compounds and serum lipids in the US adult population
Source: Lipids Health Dis. 2023 Aug 11;22:129. doi: 10.1186/s12944-023-01895-z (PMC10422774; doi:10.1186/s12944-023-01895-z)
Supplement: Supplementary file 1 — Additional File 1: Table A.1 VOC metabolites targeted in this study. Table A.2 Characteristics of the study population. Fig. A.1 Pearson correlation coefficient matrix for the VOCs visualized as a heatmap. (Red shows a positive correlation, and the intensity of the shaded squares reflects the magnitude of the correlation coefficient.) Fig. A.2 Univariate exposure–response functions and 95% credible intervals (shaded areas) for each VOC in smokers and nonsmokers with the other VOCs holding at the median. Table A.3 PIP values in BKMR. [file 12944_2023_1895_MOESM1_ESM.docx]

Table A. 1 VOC metabolites targeted in this study.

| VOC metabolites | Parent Compound | Aberration | Detection rate | LLOD  (ng/ml) |
| --- | --- | --- | --- | --- |
| 2-Methylhippuric acid | Xylene | 2MHA | 92.59% | 5.00 |
| 3- and 4-Methylhippuric acid | Xylene | 3MHA+ 4MHA | 99.60% | 8.00 |
| N-Acetyl-S-(2-carbamoylethyl)-L-cysteine | Acrylamide | AAMA | 99.90% | 2.20 |
| N-Acetyl-S-(N-methylcarbamoyl)-L-cysteine | N,N-Dimethylformamide | AMCC | 99.65% | 6.26 |
| 2-Aminothiazoline-4-carboxylic acid | Cyanide | ATCA | 96.56% | 15.0 |
| N-Acetyl-S-(benzyl)-L-cysteine | Toluene | BMA | 99.35% | 0.500 |
| N-Acetyl-S-(n-propyl)-L-cysteine | 1-Bromopropane | BPMA | 75.20% | 1.20 |
| N-Acetyl-S-(2-carboxyethyl)-L-cysteine | Acrolein | CEMA | 99.25% | 6.96 |
| N-Acetyl-S-(2-cyanoethyl)-L-cysteine | Acrylonitrile | CYMA | 86.85% | 0.500 |
| N-Acetyl-S-(3,4-dihydroxybutyl)-L-cysteine | 1,3-Butadiene | DHBMA | 99.98% | 5.25 |
| N-Acetyl-S-(2-hydroxypropyl)-L-cysteine | Propylene oxide | 2HPMA | 94.87% | 5.30 |
| N-Acetyl-S-(3-hydroxypropyl)-L-cysteine | Acrolein | 3HPMA | 99.84% | 13.0 |
| Mandelic acid | Styrene | MA | 98.59% | 12.0 |
| N-Acetyl-S-(4-hydroxy-2-butenyl)-L-cysteine | 1,3-Butadiene | MHBMA3 | 96.75% | 0.600 |
| Phenylglyoxylic acid | Ethylbenzene, styrene | PGA | 99.23% | 12.0 |
| N-Acetyl-S-(3-hydroxypropyl-1-methyl)-L-cysteine | Crotonaldehyde | HPMMA | 99.99% | 1.70 |

Table A.2. Characteristics of study population (N=1410)

| Variables | Mean (SD)/N (%)^a^ |
| --- | --- |
| Age (yr) | 48.19 (17.09) |
| Sex |  |
| Men | 729 (51.8) |
| Women | 681 (48.2) |
| Race |  |
| Non-Hispanic Black | 301 (10.1) |
| Non-Hispanic White | 602 (70.8) |
| Other race | 507 (19.2) |
| Marital status |  |
| Married or with partners | 867 (64.8) |
| Window or divorce | 253 (16.3) |
| Unmarried | 290 (18.9) |
| Education level |  |
| Less than high School | 275 (13.5) |
| High School Grad/GED or Equivalent | 299 (19.4) |
| Some College or AA degree | 428 (34.1) |
| College Graduate or above | 408 (32.9) |
| PIR |  |
| Low | 322 (14.3) |
| Middle | 572 (36.4) |
| High | 516 (49.4) |
| Smoking |  |
| Smoker | 352 (24.7) |
| Non-smoker | 1058 (75.3) |
| Drinking |  |
| No | 1065 (80.7) |
| Yes | 345 (19.3) |
| BMI (kg/m^2^) | 29.25 (6.88) |
| Creatinine (mg/dL) | 118.08 (73.05) |
| Physical activity (min) | 157.50 (265.60) |
| Energy intake (kcal) | 2242.94 (1007.11) |
| TC (mg/dL) | 193.08 (40.93) |
| TG (mg/dL) | 115.52 (66.29) |
| HDL (mg/dL) | 54.94 (16.53) |
| LDL (mg/dL) | 115.04 (35.21) |
|  |  |

^a^, Data are presented as N (weighted %) / weighted mean (SD).

| Aberration | TR | TC | LDL-C | HDL-C |
| --- | --- | --- | --- | --- |
| 2MHA | 0 | 0.05024 | 0.13388 | 0 |
| 3MHA+ 4MHA | 0.00252 | 0.00288 | 0.11788 | 0 |
| AAMA | 0.02428 | 0.0028 | 0.12452 | 0 |
| AMCC | 0.09212 | 0.0148 | 0.101 | 0.99836 |
| ATCA | 0.00248 | 0.029 | 0.197 | 0 |
| BMA | 0 | 0.00164 | 0.12068 | 0 |
| BPMA | 0 | 0 | 0.13236 | 0 |
| CEMA | 0.02004 | 0.00984 | 0.13088 | 0 |
| CYMA | 0.13152 | 0.02548 | 0.12712 | 0.0048 |
| DHBMA | 0.0006 | 0.00448 | 0.20596 | 0 |
| 2HPMA | 0 | 0 | 0.12172 | 0 |
| 3HPMA | 0.00128 | 0 | 0.12 | 0 |
| MA | 0.00592 | 0.09424 | 0.13656 | 1 |
| MHBMA3 | 0.04628 | 0.00276 | 0.11444 | 0.01448 |
| PGA | 0.00136 | 0.00248 | 0.11796 | 0 |
| HPMMA | 0.3962 | 0.00032 | 0.12504 | 0.0002 |

Table A3 PIP values in BKMR


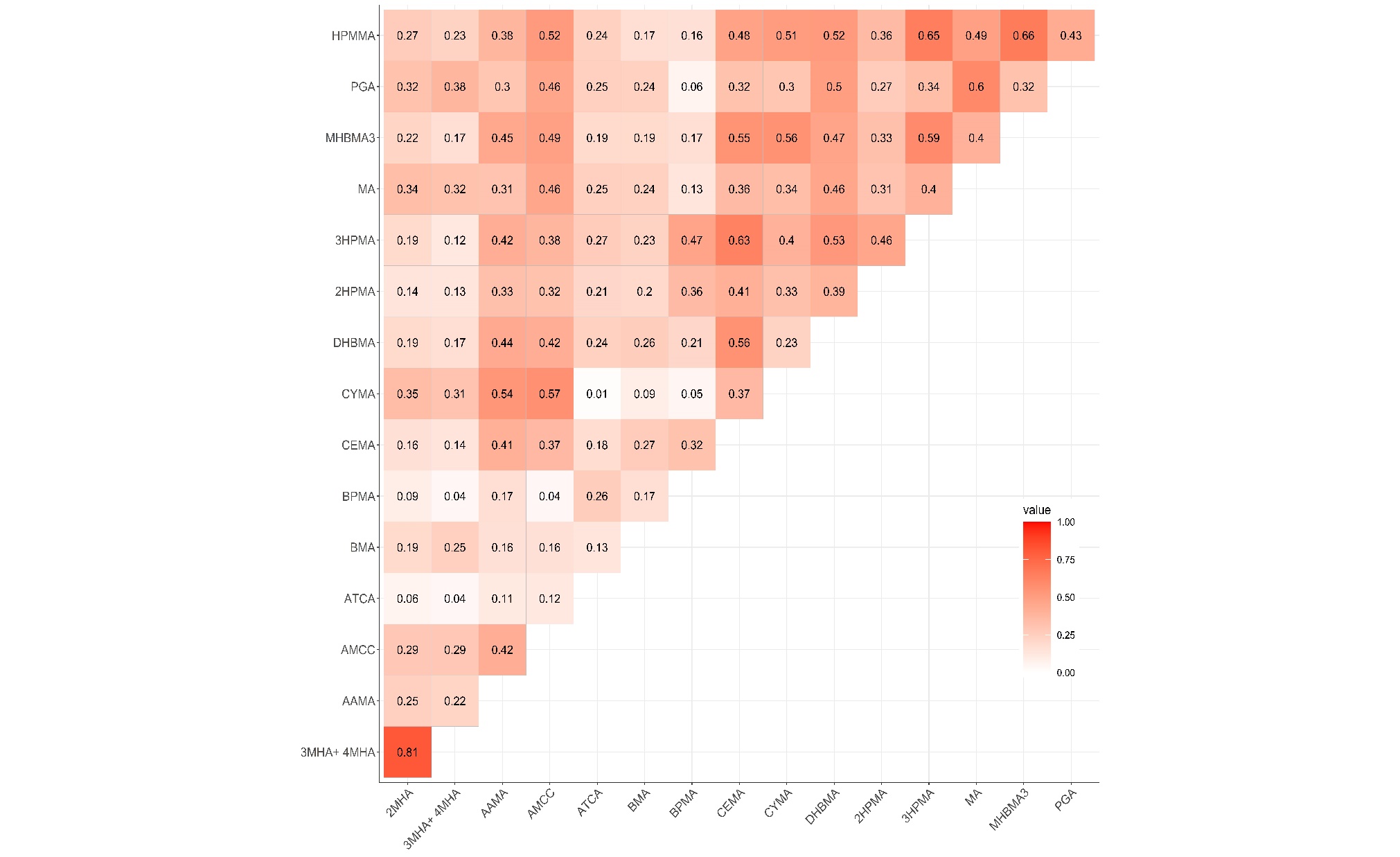


Fig. A.1 Pearson correlation coefficients matrix for the VOCs visualized as heatmap. (Red shows a positive correlation, the intensity of the shaded squares reflects the magnitude of the correlation coefficient.).


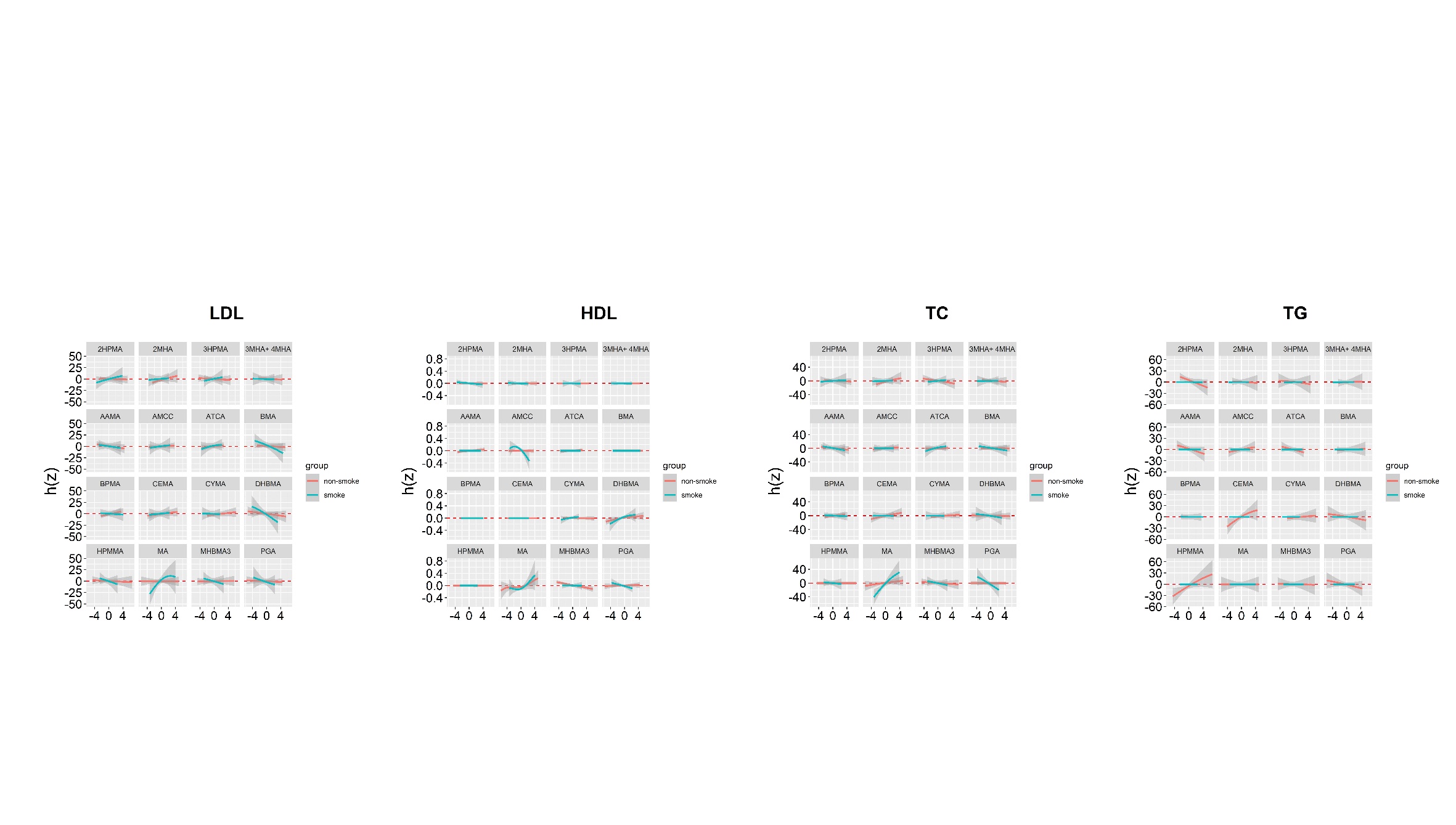


Fig. A.2 Univariate exposure–response functions and 95% credible intervals (shaded areas) for each VOC in smoker and non-smoker with the other VOCs holding at the median.


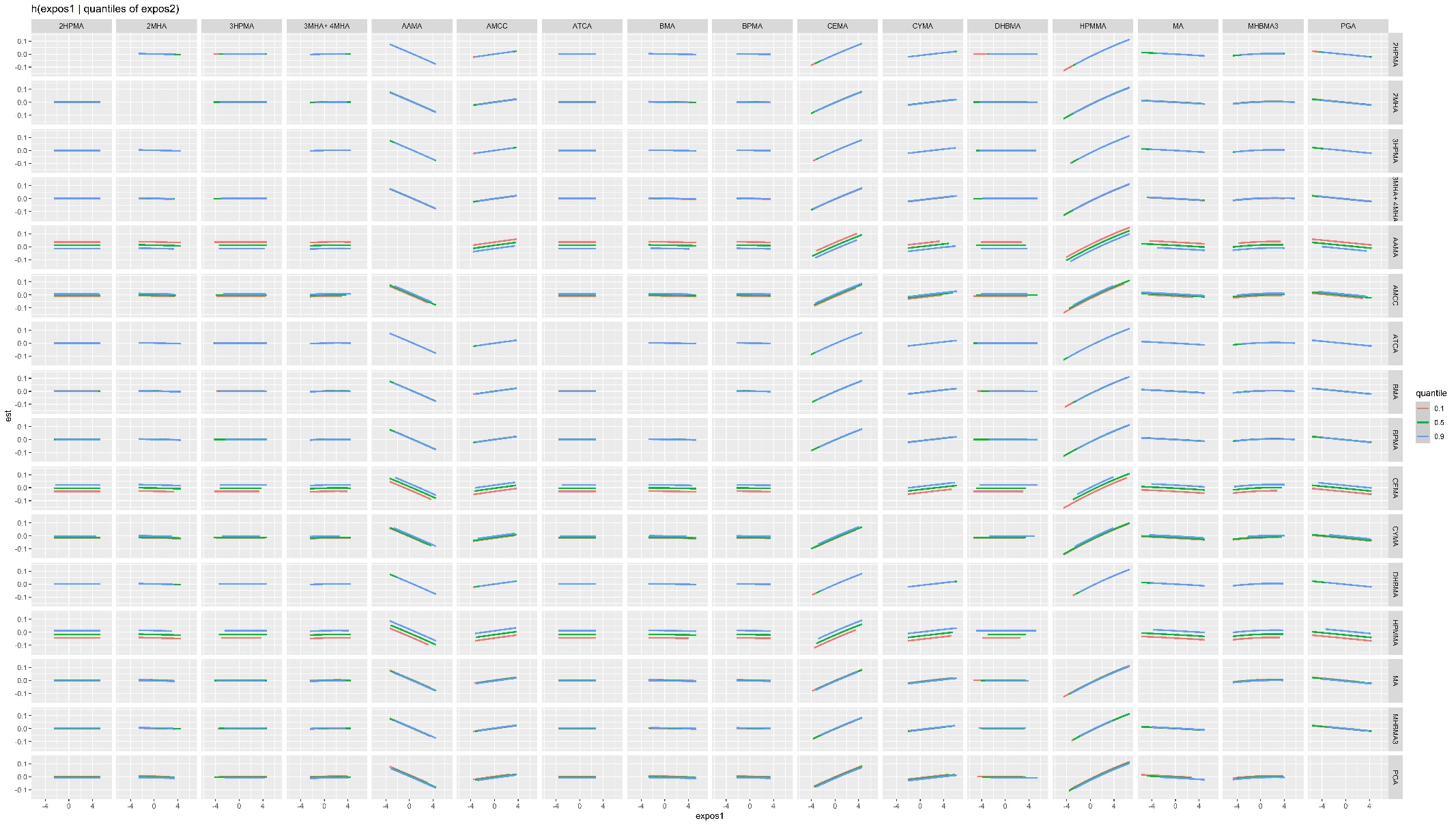


Figure A3 Interaction effect among VOCs on TC in BKMR model.


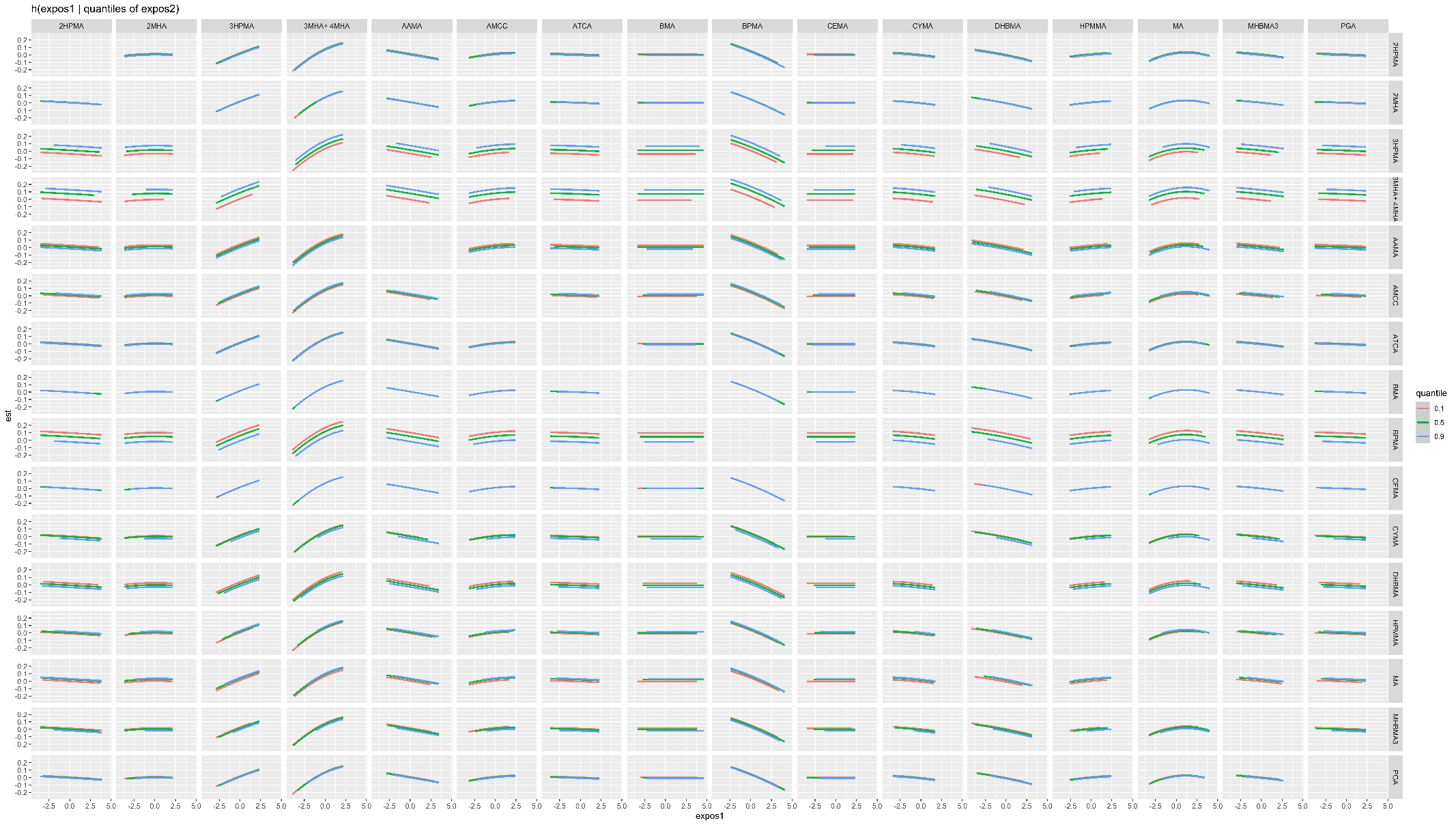


Figure A4 Interaction effect among VOCs on TG in BKMR model.


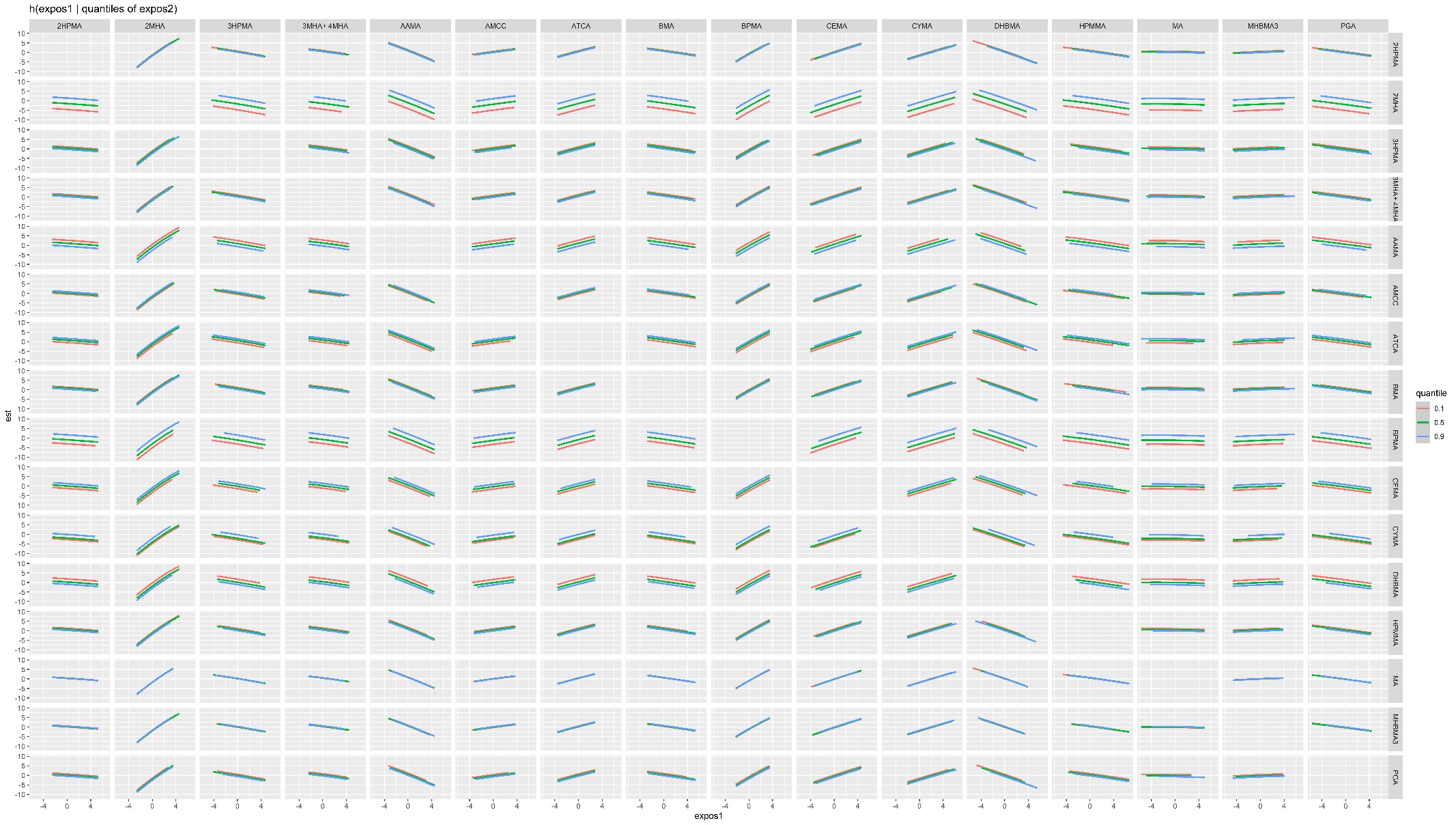


Figure A5 Interaction effect among VOCs on LDL-C in BKMR model.


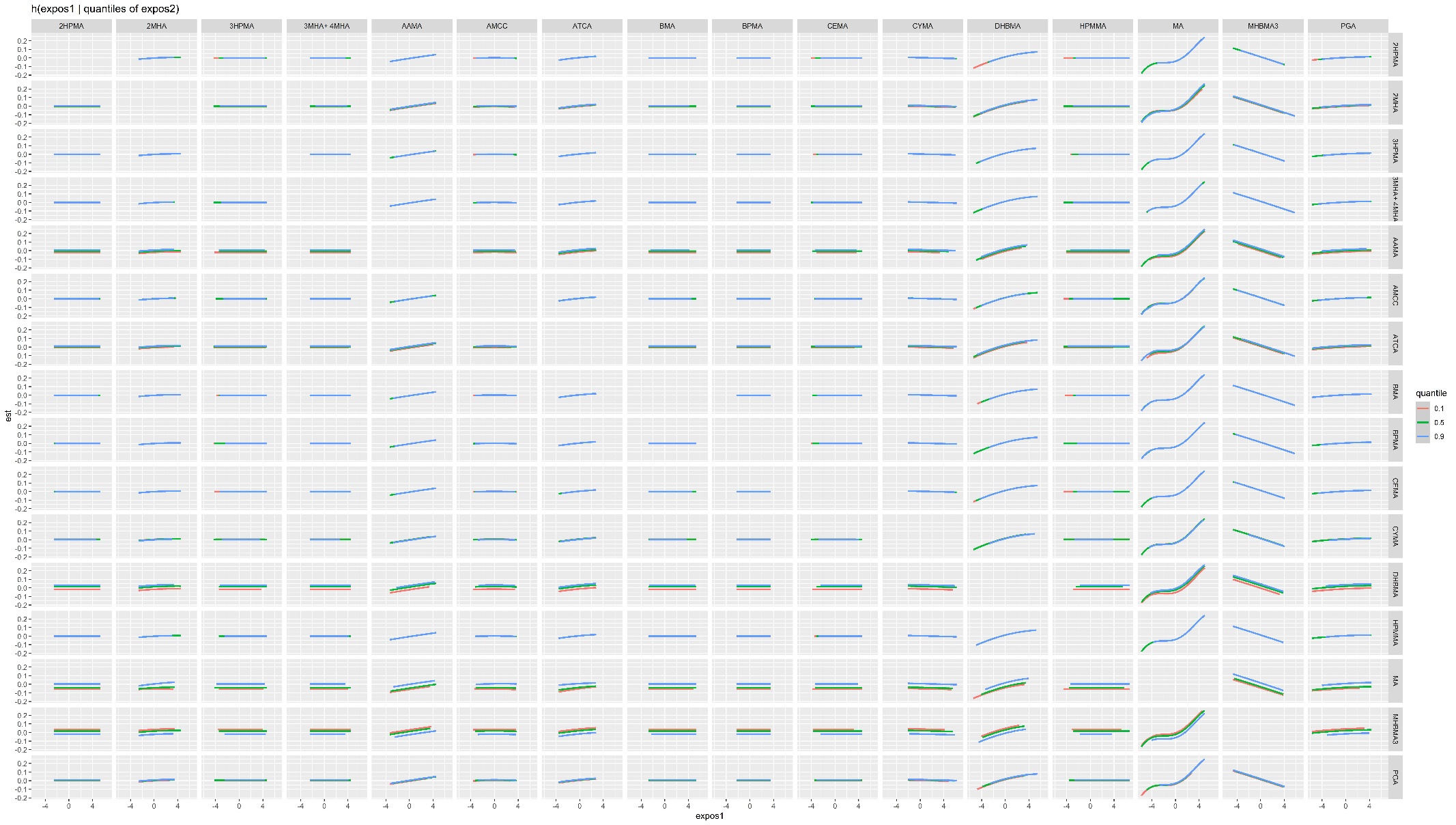


Figure A6 Interaction effect among VOCs on HDL-C in BKMR model.
